# Effect of L-Ascorbic Acid Solution Concentration on the Thermoelectric Properties of Silver Selenide Flexible Films Prepared by Vacuum-Assisted Filtration

**DOI:** 10.3390/nano12040624

**Published:** 2022-02-12

**Authors:** Yanjun Zhang, Yaxin Zhao, Rui Guo, Zengxing Zhang, Dan Liu, Chenyang Xue

**Affiliations:** State Key Laboratory of Dynamic Measurement Technology, School of Instrument and Electronics, North University of China, Taiyuan 030051, China; zhangyanjun2000@163.com (Y.Z.); zhaoyaxinnuc@163.com (Y.Z.); 18406589626@163.com (R.G.); zengxing.zhang@usn.no (Z.Z.); xuechenyang@nuc.edu.cn (C.X.)

**Keywords:** silver selenide, vacuum-assisted filtration, flexible film, thermoelectric properties

## Abstract

Currently, there are several thermoelectric materials, such as Ag_2_Te, Bi_2_Te_3_, and Sb_2_Te_3_, that have been investigated for thermoelectric applications. However, the toxicity and rarity of most of these materials make them unsuitable for practical applications. In contrast, silver selenide (Ag_2_Se) is an abundant and environment-friendly thermoelectric material. This study provides a facile synthetic approach for preparing high-performance, low-cost, and flexible Ag_2_Se thermoelectric films. Ag_2_Se nanomaterials were prepared based on the chemical template method, and the reaction solution concentration was varied to systematically investigate the effects of reaction solution concentration on the characterization and thermoelectric properties of Ag_2_Se nanomaterials. For convenience of testing, the flexible Ag_2_Se films were prepared on porous nylon membranes using vacuum-assisted filtration. The prepared thermoelectric films were tested using an X-ray diffractometer, scanning electron microscope, Seebeck coefficient tester, and Hall tester. The film prepared from the solution with the lowest concentration (18.0 mM) demonstrated the best thermoelectric performance, with a maximum power factor of 382.18 μW∙m^−1^∙K^−2^ at ~400 K. Additionally, a cold-pressing treatment could effectively enhance the electrical conductivity of the film, without damaging the substrate, as the conductivity of the film remained at 90% of the original value after 1500 bending cycles.

## 1. Introduction

The widespread use of wearable electronic devices in recent years has created a growing demand for efficient power supplies [1]. Among the various self-powered power generators [2,3], thermoelectric generators (TEGs) are ideal, maintenance-free power generators that can directly convert the temperature difference between the human body and the external environment into electrical energy, without mechanical vibration [4,5]. The conversion efficiency of TEGs depends on the thermoelectric material. Thermoelectric materials are a kind of functional material that can convert thermal and electrical energy into each other. When there is a temperature difference between the two ends of the material, the internal holes or electrons will diffuse, and a temperature difference electric potential will appear inside the material [6,7]. The key parameter that is used to describe the thermoelectric properties of the material is the thermoelectric optimum *ZT = S^2^ σT/κ*, where *S*, *σ*, *κ*, and *T* are the Seebeck coefficient, electrical conductivity, thermal conductivity, and thermodynamic temperature, respectively [8]. To obtain a high thermoelectric value, the thermoelectric material should have a high power factor (*PF = S*^2^*σ*) and a low thermal conductivity [9,10]. However, there is a strong intrinsic correlation among the Seebeck coefficient, electrical conductivity, and thermal conductivity, which makes it difficult to simultaneously optimize the electrical and thermal transport properties. Therefore, to achieve the maximum thermoelectric optimum with the combined effects of the three aforementioned parameters, the thermoelectric material should be optimized via certain methods [11].

TEGs generally consist of a combination of p-type and n-type thermoelectric materials. In the last few years, various p-type conducting polymer thermoelectric materials have been developed, such as polyaniline [12] and poly(3,4-ethylenedioxythiophenyl): poly(styrene sulfonate) (PEDOT: PSS) [13,14], which exhibit good flexibility, relatively high electrical conductivity, and low thermal conductivity. Currently, there are few studies available on n-type thermoelectric materials. To obtain higher ZT values for n-type TEGs, researchers have increasingly conducted various studies on n-type inorganic thermoelectric materials. Especially regarding flexible films [7,15,16]. Flexibility is generally defined as the ability to withstand a certain degree of extrusion and deformation without loss of performance. For instance, Jie et al. used a novel fiber-assisted cold-pressing method to prepare Ag_2_Te thin films with a power factor of 192 μW∙m^−1^∙K^−2^ [17]. Nuthongkum et al. prepared Bi_2_Te_3_ thin films with a power factor of 1550 μW∙m^−1^∙K^−2^ using the magnetron sputtering technique [18]. Dong et al. proposed a facile fabrication method to modify a cellulose paper with inorganic thermoelectric powders (Bi_2_Te_3_ and Sb_2_Te_3_) via vacuum filtration [19], which can produce an output voltage of 41.2 mV at a temperature difference of 50 K. However, Te elements are rare and toxic, making them unsuitable for practical applications in wearable devices [20,21,22]. Silver selenide (Ag_2_Se), as an electron-crystal phonon-liquid n-type material, is environment-friendly and abundant. It exhibits high electrical conductivity and low thermal conductivity at room temperature, and, therefore, it has emerged as a promising n-type material in recent years for thermoelectric applications. For example, Mi et al. investigated the effects of excess Se on the thermoelectric properties of Ag_2_Se [23], where a maximum thermoelectric ZT of 0.85 was obtained for Ag_2_Se_1.06_. Zhou et al. prepared unsupported thermoelectric films of polyvinylidene fluoride (PVDF)/Ag_2_Se via a drop coating method [24], and the maximum power factor obtained was 189.02 μW∙m^−1^∙K^−2^ when the mass ratio of PVDF to Ag_2_Se was 1:9.5. Although the addition of PVDF significantly improved the flexibility and stability, it also reduced the thermal conductivity of the thermoelectric film, owing to the insulating nature of PVDF. Perez et al. prepared Ag_2_Se thin films using pulsed mixed-reaction magnetron sputtering, with a power factor value of approximately 2440 μW∙m^−1^∙K^−2^ [25]. However, magnetron sputtering equipment is expensive, and the sputtering target always produces inhomogeneous erosion during the sputtering process. In addition, the sputtering targets are easy to damage and have a low utilization. In recent years, some researchers have used vacuum-assisted filtration methods to prepare flexible films, which reduces material waste relative to drop coating [24] and is without the expensive equipment costs relative to magnetron sputtering [25]. We previously prepared Ag_2_Se/polyvinylpyrrolidone (PVP) composite films using vacuum-assisted filtration, whose maximum power factor was 16.18 μW∙m^−1^∙K^−2^ at 320 K [26]. However, PVP as an insulating polymer led to a significant decrease in the conductivity of the thermoelectric films and reduced their thermoelectric properties.

In this study, we propose a simple method to prepare Ag_2_Se/PVP composite films. Briefly, Ag_2_Se nanorods were prepared using the chemical template method, and flexible thermoelectric films were then prepared on porous nylon membranes using vacuum-assisted filtration. The effects of different reaction solution concentrations on the thermoelectric properties and flexibilities of the flexible Ag_2_Se films were systematically investigated. Furthermore, a cold-pressing treatment technique was used to prepare the films, which can effectively enhance the electrical conductivity of the films without damaging the substrates and can be applied to most substrates.

## 2. Materials and Methods

### 2.1. Materials

Anhydrous ethanol, ethylene glycol (EG), l-ascorbic acid, β-cyclodextrin, selenium dioxide (SeO_2_), and silver nitrate (AgNO_3_) used in the experiments were purchased from Aladdin Industrial Corporation (Shanghai, China). The porous nylon filter membrane (average pore size of approximately 0.22 μm) was purchased from Wuxi Yatai United Chemical Co., Ltd. (Wuxi, China). All the reagents were used directly, without purification.

### 2.2. Preparation of Ag_2_Se Nanorods and the Flexible Thermoelectric Film

All the containers and tools were cleaned ultrasonically before use. First, 1 g of SeO_2_ and 1 g of β-cyclodextrin were added to deionized water, and the mixture was stirred magnetically until completely dissolved. Then, a solution of 4 g of l-ascorbic acid was added to deionized water with magnetic stirring until complete dissolution, which was then added slowly and dropwise to the former solution. The mixed solution was then magnetically stirred for 4 h at room temperature for completion of the reaction. The supernatant was then removed by centrifugation at 8500 rpm for 5 min and washed twice with alternate centrifugation with deionized water and anhydrous ethanol. Finally, the product was dispersed in anhydrous ethanol and left for 36 h, before proceeding to the next step of the experiment. The precipitate from the above solution was transferred to 100 mL of EG and dispersed via sonication. Based on the molar ratio of Ag and Se, a certain amount of AgNO_3_ was dissolved in the 100 mL EG, and this solution was then slowly added dropwise to the above precipitate solution. The molar mass ratio of l-ascorbic acid to AgNO_3_ was 3:1. After the completion of the reaction by magnetic stirring for 4 h at room temperature, the supernatant was removed by centrifugation at 8500 rpm for 5 min and washed twice with deionized water and anhydrous ethanol alternately. Finally, the precipitated Ag_2_Se nanomaterial was collected by drying at 60 °C under a nitrogen atmosphere.

To verify the effects of solution concentration on the thermoelectric properties of Ag_2_Se nanomaterials during the experiments, control experiments were conducted by varying only the amounts of deionized water for the complete reaction of SeO_2_, β-cyclodextrin, and l-ascorbic acid, which were 166 mL, 250 mL, 400 mL, and 500 mL. The prepared films were named according to the solution concentration, as 54.0 mM, 36.0 mM, 22.5 mM, and 18.0 mM, respectively.

**Figure 1:**
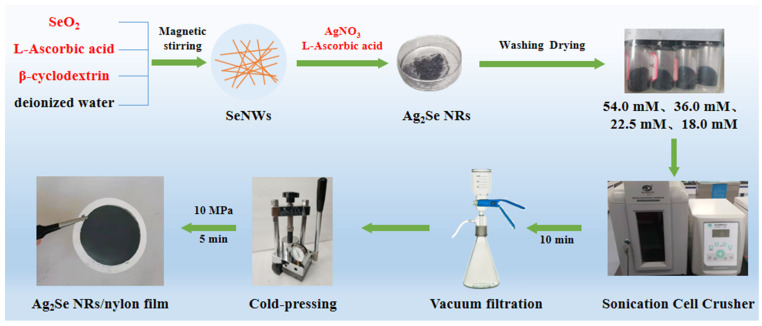
Schematic representation of the preparation of the Ag_2_Se nanomaterials and the flexible thermoelectric films.

For preparing the films, 0.03 g of each powder was ultrasonically dispersed (JY96-IIN, SCIENTZ) in 30 mL of ethanol, and deposited onto a porous nylon membrane by vacuum-assisted filtration. The as-prepared films were dried at 60 °C for 3 h under a nitrogen atmosphere and then cold-pressed for 5 min at 10 MPa. The preparation process of the Ag_2_Se nanomaterials and flexible thermoelectric films is shown in Figure 1.

### 2.3. Testing and Characterization

The physical phase structural composition of the Ag_2_Se nanomaterials was determined using X-ray diffraction (XRD; Hoyuan Instruments, DX-2700, Dandong, China). The surface and cross-sectional thicknesses of the flexible thermoelectric films were determined using scanning electron microscopy (SEM; ZEISS, JSM-7900F, Tokyo, Japan). The Seebeck coefficient and conductivity were determined using a Seebeck coefficient tester (Linseis, LSR-3, Selb, Germany) based on the standard four-probe method. The carrier concentration and mobility were determined using a Hall tester (Linseis, HCS, Selb, Germany) based on the Vanderbilt method, and the measurement error was about ±5%. An appropriate amount of thermoelectric film was cut and bent using a metal rod 8 mm in diameter, to test the change in conductivity with increasing bending time.

## 3. Results and Discussion

**Figure 2:**
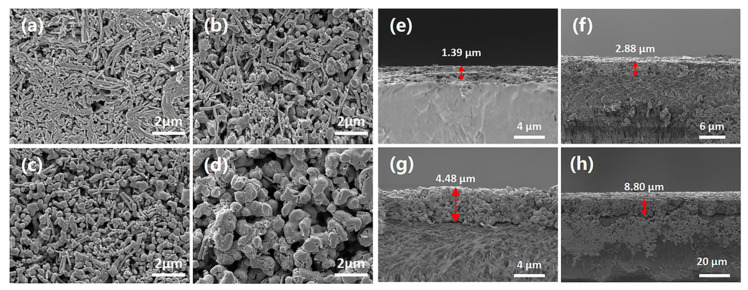
Scanning electron microscopy (SEM) images of the surface morphologies (**a**) 18.0 mM, (**b**) 22.5 mM, (**c**) 36.0 mM, (**d**) 54.0 mM and cross-sections (**e**) 18.0 mM, (**f**) 22.5 mM, (**g**) 36.0 mM, (**h**) 54.0 mM of four flexible Ag_2_Se thermoelectric films.

The surface morphologies and cross-sections of the four flexible Ag_2_Se thermoelectric films are shown in Figure 2. It can be seen from the surface morphology that the structures of the Ag_2_Se nanomaterials with various reaction solution concentrations are very different. The 18.0 mM film mostly consists of one-dimensional rod-like structures, with non-uniform lengths. After cold-pressing, the rod-like structures are closely packed and the film surface becomes relatively dense. With the increase in concentration, zero-dimensional granular nanostructures begin to appear, and the amount of one-dimensional rod-like nanostructures decreases gradually. Zero-dimensional granular nanostructures are predominant in the reaction solution with a 36.0 mM concentration. With an increase in the reaction solution concentration from 36.0 mM to 54.0 mM, the size of the zero-dimensional granular nanostructures began to increase and the one-dimensional linear nanostructures disappeared. Therefore, the lateral size of the nanomaterials can be easily controlled by adjusting the concentration of the solution.

SEM was performed on the cross-sections of the four films, to compare the effects of different reaction solution concentrations on the surface cross-sections of the thermoelectric films during the experiments and to facilitate the subsequent testing of the thermoelectric properties. From Figure 2, although the same mass of Ag_2_Se nanomaterials was used to prepare the flexible films for the experiments, the thicknesses of the four flexible thermoelectric films differed significantly. The average measured thicknesses of the films were 1.39 μm, 2.88 μm, 4.48 μm, and 8.8 μm, for the 18.0 mM, 22.5 mM, 36.0 mM, and 54.0 mM films, respectively.

**Figure 3:**
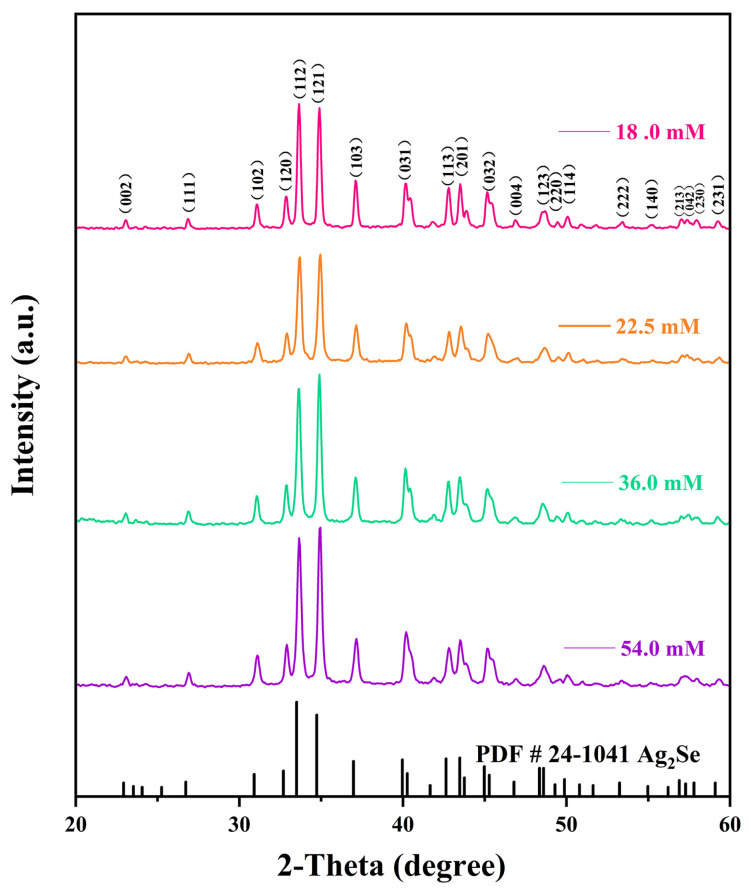
X-ray diffraction (XRD) patterns of the four flexible Ag_2_Se thermoelectric films.

The effects of different reaction solution concentrations on the physical phase structures of the Ag_2_Se nanomaterials were investigated using XRD, and the results are shown in Figure 3. The diffraction peaks of all four Ag_2_Se nanomaterials correspond to those of the standard card PDF#24-1041, indicating that the target products were successfully prepared by this experimental scheme. Moreover, the peaks are sharp, indicating that the products have good crystallinity. In addition, diffraction peaks of other substances were not found, which implies that the prepared products are pure. It can further be observed from the peaks at (112) and (121) that the nanomaterials gradually and selectively grew in the direction of the peak (112) as the concentration of the reaction solution decreased. Comparing the structural morphologies of the nanomaterials in Figure 2, we noted a gradually increasing amount of one-dimensional nanowires, which replaced the zero-dimensional nanoparticles when the concentration of the reaction solution was decreased.

**Figure 4:**
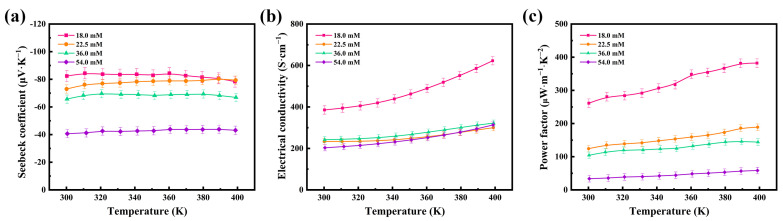
Temperature dependence of the (**a**) Seebeck coefficients, (**b**) electrical conductivities, and (**c**) power factors of the four Ag_2_Se flexible thermoelectric films.

The thermoelectric properties of the flexible thermoelectric films were tested at different concentrations, and Figure 4 shows the variations in the Seebeck coefficient (*S*), electrical conductivity (*σ*), and power factor (*PF*) of the 54.0 mM, 36.0 mM, 22.5 mM, and 18.0 mM films in the temperature range of 300–400 K. Figure 4a shows the variation in the Seebeck coefficients of the four flexible thermoelectric films with temperature. The Seebeck coefficients of all four flexible thermoelectric films are negative, indicating that Ag_2_Se is an n-type semiconductor and that the change in the concentration of the reaction solution does not affect the intrinsic conductivity type of Ag_2_Se. The Seebeck coefficients remain constant at temperatures of 300–400 K, demonstrating the high stability of the prepared Ag_2_Se nanomaterials near room temperature. Among the four films, the Seebeck coefficient of the 18.0 mM film was the highest, with 82.33 μV∙K^−1^ near room temperature. The Seebeck coefficient decreased gradually with the increasing concentration of the reaction solution. The 54.0 mM film had the lowest Seebeck coefficient, at 40.64 μV∙K^−1^ near room temperature, which is half of that of the 18.0 mM film.

Figure 4b shows the variation in the electrical conductivities of the four flexible thermoelectric films with temperature. The films exhibited semiconductor properties and their electrical conductivities gradually increased with temperature. The electrical conductivity of the 18.0 mM film was the maximum, with 385.42 S∙cm^−1^ near room temperature. This is because the nanomaterials are one-dimensional rods, and the electron scattering is significantly weakened by the close contact of the nanorods after cold-pressing. The electrical conductivity of the 18.0 mM film increases with temperature, reaching 622.77 S∙cm^−1^ at 400 K. The electrical conductivity of the 54.0 mM film was the minimum, at 203.01 S∙cm^−1^ near room temperature, and it increased with temperature, becoming 313.32 S∙cm^−1^ at 400 K.

Under the condition that the thermal conductivity can be temporarily disregarded when the film thickness is small, it can be concluded that the thermoelectric properties of the flexible thermoelectric films primarily depend on the power factor *PF* (*PF = S*^2^*σ*), as can be seen from the dimensionless thermoelectric optimum *ZT* (*ZT = S*^2^ *σT/κ*). Figure 4c shows the variation in the power factor of the four flexible thermoelectric films with temperature. The power factor of the 18.0 mM film was the maximum, with 261.26 μW∙m^−1^∙K^−2^ at room temperature, and it increased up to 382.18 μW∙m^−1^∙K^−2^ with increasing temperature. Therefore, this film showed the best thermoelectric performance, owing to the combined effect of the Seebeck coefficient and electrical conductivity. Due to the limited experimental conditions, experiments with smaller concentrations of reaction solutions were not conducted and will be performed in future experiments.

**Figure 5:**
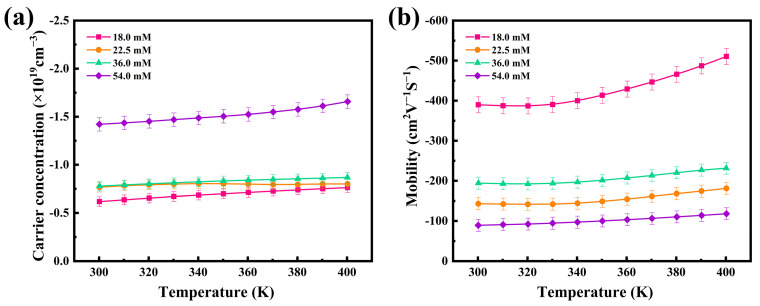
Temperature dependence of the (**a**) carrier concentrations and (**b**) mobilities of the four Ag_2_Se flexible thermoelectric films.

Based on the recorded thermoelectric properties, the internal mechanism of thermoelectric performance enhancement of the thermoelectric films was further investigated, and the carrier concentration (*n*) and mobility (*μ*) of four flexible thermoelectric films were evaluated using a Hall tester. The results are shown in Figure 5. The electrical conductivities and Seebeck coefficients are closely related to the internal carrier concentration and mobility of the thermoelectric materials, through the following Equations (1) and (2):

(1) $$S=\frac{8\pi^{2}k_{B}^{2}}{3eh^{2}}m^{*}T{\left(\frac{\pi }{3n}\right)}^{2/3}$$

(2) σ=neμ

where m^*^ is the carrier effective mass and *h* and *k_B_* are the Planck and Boltzmann constants, respectively. From Figure 5, can be seen a slow increase in the carrier concentrations and mobilities between 300 K and 400 K. The carrier concentration has a significant effect on the Seebeck coefficient according to Equation (1), under the condition that the variation of the effective mass of carriers is not considered. As the carrier concentration increases, the value of the Seebeck coefficient decreases. The results, as depicted in Figure 5a, show that the carrier concentration of the 18.0 mM film was 0.62 × 10^19^ cm^−3^, and that of the 54.0 mM film was 1.42 × 10^19^ cm^−3^ at room temperature, which explains the observed difference in Seebeck coefficients at the same temperature, as observed in Figure 4. According to Equation (2), the electrical conductivity change of the film is affected by both the carrier concentration and mobility. In Figure 5, both the carrier concentration and mobility increase with temperature. Accordingly, there was a gradual increase in the electrical conductivity of the film, as also shown in Figure 4. At room temperature, the mobility of the 18.0 mM film was 389.71 cm^2^∙V^−1^∙S^−1^ and that of the 54.0 mM film was 89.14 cm^2^∙V^−1^∙S^−1^. Owing to the synergistic effects of its superior carrier concentration and mobility, the 18.0 mM film had the best conductivity among the films, as calculated by Equation (2).

**Figure 6:**
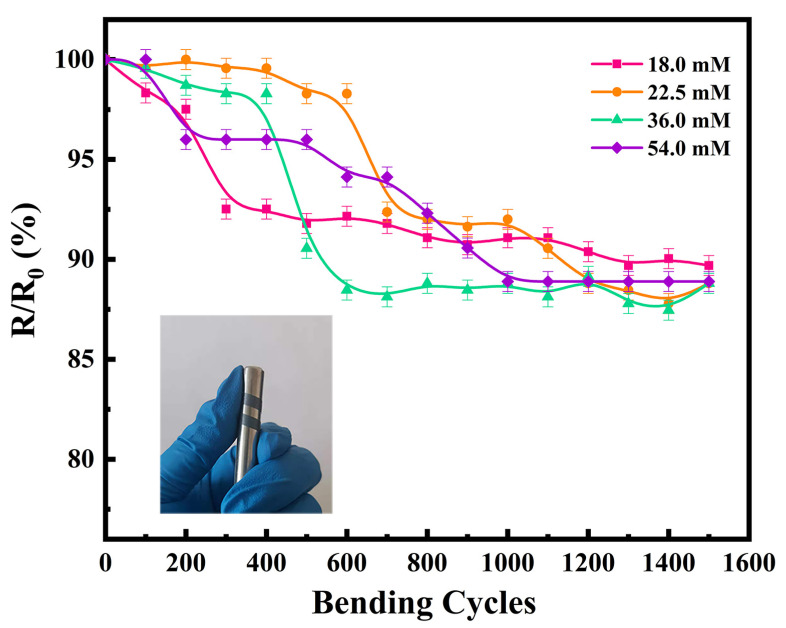
Flexibilities of the four Ag_2_Se flexible thermoelectric films (physical test image is attached).

In addition to the thermoelectric properties, flexibility is another key factor to be considered for practical application of TEGs. An appropriate amount of thermoelectric film was cut and bent using a metal rod 8 mm in diameter, to test the change in electrical conductivity with an increasing number of bending cycles. The variation in the electrical conductivities of the flexible thermoelectric films with the number of bending cycles is shown in Figure 6. The electrical conductivity of all four flexible thermoelectric films decreased to a certain extent with an increase in the number of bending cycles. The electrical conductivity after 1500 bending cycles was approximately 90% of the original value, which proves that the prepared films have good flexibility. In our previous study, the electrical conductivity of the film decreased to 68.8% after 1500 bending cycles. Therefore, the films prepared in the present study are more flexible than those we reported previously [26]. This is likely because we used a cold-pressing treatment in the present work, which caused the Ag_2_Se nanomaterials to bond with each other; thus, ensuring the electrical conductivity of the film was maintained over continuous bending cycles. Consequently, cold-pressing is a cost-effective method to prepare films without damaging the substrate, while effectively enhancing the electrical conductivity of the film.

## 4. Conclusions

In this study, nylon-based Ag_2_Se flexible thermoelectric films were prepared by vacuum-assisted filtration, and their thermoelectric properties and flexibilities were systematically investigated at different concentrations. The Ag_2_Se nanorods were synthesized easily and efficiently via a chemical template method using Se nanorods. The test results show that the 18.0 mM film, prepared from a solution with the lowest concentration, had the best thermoelectric performance, exhibiting a maximum power factor of 382.18 μW∙m^−1^∙K^−2^ near 400 K. In addition, the cold-press treatment could effectively enhance the electrical conductivity of the film, without damaging the substrate, as the electrical conductivities of the films prepared by the cold-press treatment decreased to only 90% of the original value after 1500 bend cycles. This provides a simple and efficient approach for the preparation and application of environmentally friendly flexible Ag_2_Se thermoelectric devices for the development of wearable electronics.

## Data Availability

The data presented in this study are available on request from the corresponding author.
